# The Autologous Hematopoietic Stem Cells Transplantation Combination-Based Chimeric Antigen Receptor T-Cell Therapy Improves Outcomes of Relapsed/Refractory Central Nervous System B-Cell Lymphoma

**DOI:** 10.1155/2022/2900310

**Published:** 2022-11-29

**Authors:** Fei Xue, Peihao Zheng, Rui Liu, Shaomei Feng, Yuelu Guo, Hui Shi, Haidi Liu, Biping Deng, Teng Xu, Xiaoyan Ke, Kai Hu

**Affiliations:** ^1^Department of Adult Lymphoma, Beijing Boren Hospital, Beijing, China; ^2^Cytology Laboratory, Beijing Boren Hospital, Beijing, China

## Abstract

**Objective:**

The objective is to explore the effectiveness and safety of CAR T-cell therapy in advanced relapsed/refractory central nervous system B-cell lymphoma and compare the impact of autologous stem cell transplantation (ASCT) plus CAR T-cell therapy versus sequential CART therapy on the survival of patients.

**Methods:**

The retrospective analysis was based on the data of 17 patients with advanced relapsed/refractory central nervous system B-cell lymphoma. Bridging chemotherapy was applied before CAR T-cell infusion to further reduce the tumor burden. For patients with autologous hematopoietic stem cell successful collection, CD19/20/22CAR T-cell immunotherapy following ASCT was performed with the thiotepa-containing conditioning regimen, while sequential CD19/CD20/CD22CAR T-cell therapy was applied. For lymphodepletion, patients received bendamustine or fludarabine monotherapy or fludarabine combined with cyclophosphamide pre-CART-cell infusion.

**Results:**

Out of the 17 patients, 8 completed ASCT plus CART cell therapy, while 9 patients completed CART cell alone therapy. In efficacy assessment at 3 months after infusion, the objective response rate (ORR) was 12/17 (71%) and the complete response rate (CRR) was 11/17 (65%). The CRR of the ASCT group and non-ASCT was 100% and 44.4%, respectively (*P* < 0.01). The median progression-free survival was 16.3 (2.6–24.5) months, and the median overall survival was 19.3 (6–24.5) months. Patients who underwent ASCT plus CART cell therapy had significantly longer PFS (*P* < 0.01) and OS (*P* < 0.01). Grade 3 or higher immune effector cell-associated neurologic toxicity syndrome (≥grade 3 ICANS) and cytokine release syndrome (≥grade 3 CRS) events occurred in 29% and 41% of the patients, respectively. No treatment-related death occurred.

**Conclusion:**

The CAR T-cell therapy could augment its efficacy in the treatment of advanced relapsed/refractory CNS B-cell lymphoma, while ASCT in combination with CART can induce durable responses and OS with a manageable side effect.

## 1. Background 

Central nervous system (CNS) lymphoma includes primary central nervous system lymphoma (PCNSL) and secondary central nervous system lymphoma (SCNSL), both of which are usually treated with the regimen of aggressive high-dose methotrexate (MTX) [1, 2] or thiotepa-based induction chemotherapy and autologous hematopoietic stem cell transplantation (ASCT) or whole-brain radiotherapy consolidation treatment [3, 4]. The complete remission (CR) rate of PCNSL patients has been reported to be approximately 45% [5–7]. However, approximately 35%–60% of patients relapse within 1-2 years, and nearly 10%–15% of patients are not sensitive to therapy [8]. The prognosis of patients with SCNSL is even worse, as long-term survival can be achieved in less than 20% of patients [9]. Although targeted drugs such as Bruton tyrosine kinase inhibitors (BTKis), Lenalidomide, and programmed death-1 inhibitors have improved the outcomes of central nervous system (CNS) lymphoma (the best CR rate 86%), patients tend to develop drug resistance rapidly, and the prognosis of these suffers remains poor [9]. Refraction and recurrence are the major causes of treatment failure in patients with CNS lymphoma [10–13]. Nevertheless, there is no consensus in the standard treatment for relapsed/refractory CNS (r/r CNS) lymphoma, currently. Therefore, it is a pressing issue that searching for a more effective treatment regimen for these challenging patient population.

Chimeric antigen receptor-T cell (CART) therapy can effectively improve the complete remission (CR) rate of relapsed/refractory malignant B-cell tumors (range from 39% to 58%) and progression-free survival (median progression-free survival of 5.9 months) [14–18]. Yet, concerns for potential life-threatening neurotoxicity of CART cells and immune privileged of central nervous system, patients with r/r CNS lymphoma are excluded from pivotal cohort studies, and little is known about its effectiveness and treatment-related toxicities [14, 19]. Recently, several studies [20–24] (ranging from case reports or series to cohort studies) reported on the controllability of neurological toxicities and the effectiveness of CART cells in treating r/r CNS B-cell lymphoma. A retrospective study with eight patients diagnosed with secondary CNS B-cell lymphoma treated by CD19CART cells showed encouraging efficacy and manageable adverse events. A total of 4 patients were response to treatment and no patient experienced greater than grade-1 neurotoxicity [22]. Another prospective cohort study related to CAR T-cell immunotherapy in patients with relapsed PCNSL demonstrated that the overall response rate (ORR) is 58% (7/12), and the rate of ICANS is 50% but severe neurotoxicity (≥grade 3) 8% (1/12) [23]. These findings suggest that it is possible to treat r/r CNS B-cell lymphoma by CAR T-cell immunotherapy, but the duration of the responses was relatively short (median PFS ranging from only 3 months to 4.4 months) [25, 26]. Hence, to improve the poor outcome of low long-term remission rate, investors resort to combination with consolidation therapy.

For CNS lymphoma, autologous stem cell transplantation (ASCT) and whole-brain radiation therapy (WBRT) have been used as standard consolidation treatments in the past [27]. However, patients with WBRT alone were prone to disabling cognitive dysfunction and devastating consequences on the quality of life [27, 28]. In the prospective study, patients with PCNSL were treated by cranial irradiation following chemotherapy and the incidence of severe neurologic toxicity was 15% [29]. WBRT probably increases the neurotoxicity of CART cells for treating CNS lymphoma. Instead of WBRT, combination with ASCT is naturally selected, this combination therapy has been applied to relapsed/refractory multiple myeloma and non-CNS lymphoma, and conditioning regime pre-ASCT can deeply deplete lymphocytes inhibiting the function of CART cells [30–32]. Recently, CAR T-cell immunotherapy following autologous stem cell transplantation (ASCT) for central nervous system lymphoma has been reported [26, 33]. The overall response rate (ORR) is nearly 82%, and the complete remission rate (CRR) is approximately 55%. The median durable response achieved a relatively longer at 14.03 months [33]. The incidence of severe immune effector cell-associated neurologic toxicity was 8%. However, it is not available for patients who cannot tolerate the toxicity of chemotherapy or without hematopoietic stem cells. In recent years, separate CAR T-cell immunotherapeutic avenues such as “Dual-Target” and “cocktail” CAR T-cell therapies are also administrated to attain ongoing complete remission [20, 26]. The patient in the former report continued CR for more than 17 months, but the median PFS in the latter study was only 3 months, which appeared to be a shorter term than ASCT plus CART. However, very few subjects were included. Therefore, we retrospectively investigated the effectiveness and safety of CART cells in treating 17 patients with r/r CNS B-cell lymphoma in the real-world and firstly compared the impact of ASCT plus CAR T-cell therapy versus sequential multitargeted (CD19, CD20, and CD22) CAR T-cell on durable remission.

## 2. Materials and Methods

### 2.1. Participant Population

**Figure 1:**
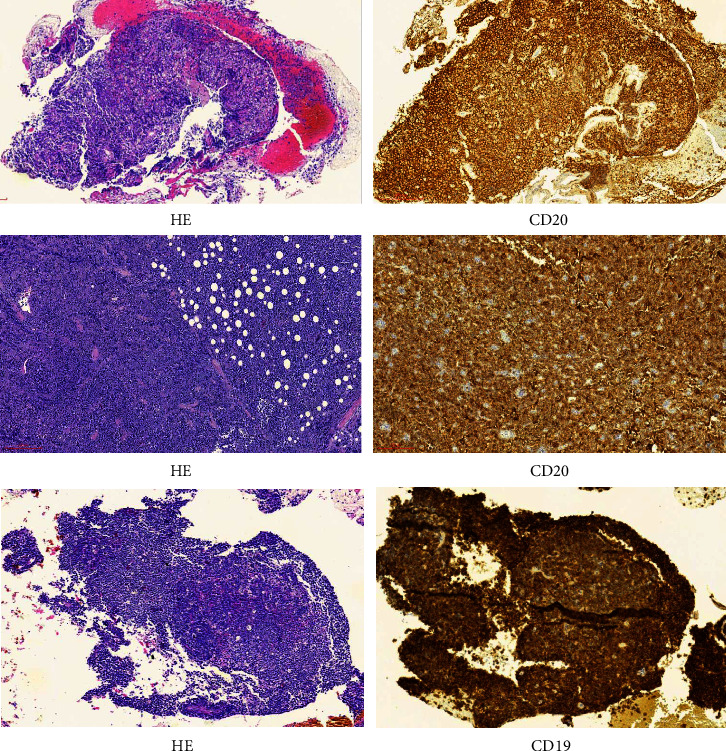
Representative images of three patients with diffuse large B-cell lymphoma with central nervous system involvement are demonstrated (H & E, original magnification x100 and immunohistochemistry, original magnification x100).

Data from 17 patients with advanced r/r CNS B-cell lymphoma enrolled in the clinical- trial “Different B cell-targeted CART sequential infusion for adult patients with relapsed/refractory aggressive B-cell lymphoma (Clinicaltrials.gov registry: ChiCTR1900020980)” in the Beijing Boren Hospital between October 1, 2018, and October 1, 2020, were retrospectively analyzed. On the basis of the 2016 World Health Organization (WHO) guidelines and the diagnosed criteria of SCNSL [34–36], the diagnosis of CNS B-cell lymphoma by stereotactic biopsy and/or lumbar puncture for immunochemistry (IHC) (Figure 1) and/or flow cytometry (FCM) has been confirmed. An imaging examination was performed to clarify the lesion site. Of the 17 patients, 10 had brain parenchymal involvement, 4 had cerebrospinal fluid (CSF) involvement, and 3 had both brain parenchymal and CSF involvement. This study was approved by the Ethics Committee of the Beijing Boren Hospital, and all patients signed an informed consent form.

### 2.2. Procedures

Peripheral blood mononuclear cells (PBMNCs) were isolated from the eligible patients, and CD3^+^ T lymphocytes were separated by using antigen-coated immunomagnetic beads. CD19/CD20/CD22 expression in tumor tissues was identified by IHC and FCM, which was the basis for selecting targets for CART cells. The second generation anti-CD19, CD20, and CD22-41BB-CAR lentiviral vector was constructed to transfect purified CD3^+^ T cells to prepare CART cells. The detailed processes have already been described in previous studies [37–39].

Bridging chemotherapy was permitted prior to CAR T-cell transfusion to reduce tumor burden (for patients with CSF involvement, an intrathecal injection of 15 mg methotrexate, 50 mg cytarabine, and 5 mg dexamethasone, twice per week was performed until the minimal residual disease of the CSF showed negative by FCM). For patients with a response to chemotherapy, autologous hematopoietic stem cells were mobilized by granulocyte colony-stimulating factors and collected. Patients with successful stem cell collection received ASCT in combination with CAR T-cell therapy with the TEAM (thiotepa 5 mg/kg, d-8 to d-7; VP-16 200 mg/m^2^·d, d-6 to d-3;Ara-C 200 mg/m^2^·d, d-6 to d-3; and melphalan 140 mg/m^2^·d, d-2) or BEAM (BCNU 300 mg/m^2^, d-6; VP-16 200 mg/m^2^·d, d-5 to d-2, Ara-C 200 mg/m^2^, q12 h, d-5 to d-2; and Mel 140 mg/m^2^, d-1)-based conditioning regimen. The detailed dosages were adjusted according to the fundamental status and tolerance of the patients. Taking the date of CART transfusion as day 0, ASCT was transfused on day-1.

**Figure 2:**
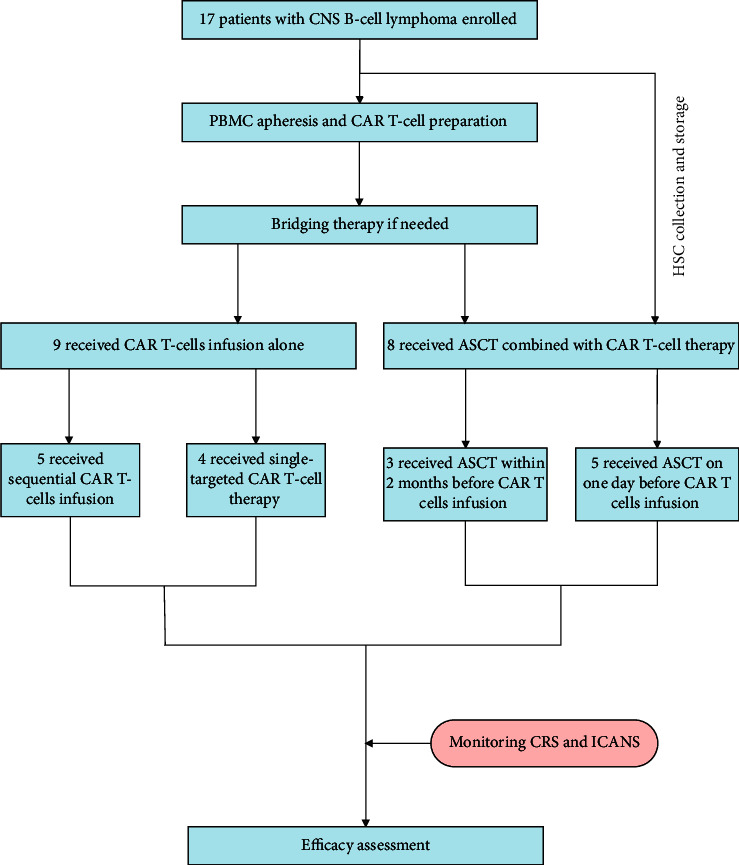
Flow diagram of the 17 patients underwent treatment.

For patients with insufficient/without autologous stem cells, sequentially different (CD19, CD20, and CD22) CART cell therapy was performed, and the sequential interval between different targeted CAR T-cell infusions was within 3 months. For all the patients, bendamustine (90–100 mg/m^2^) or fludarabine (25–30 mg/m^2^, d-3 to d-1) monotherapy or in combination with cyclophosphamide (CTX, 250 mg/m^2^, d-4 to d-2) was administrated for lymphocyte clearing prior to CART cell transfusion (Figure 2).

A multicolor flow cytometer (FACS Calibur, BD, USA) was used to detect the CAR T-cell concentration in the blood and cerebrospinal fluid (CSF). Enzyme-linked immunosorbent assay (ELISA) was used to dynamically monitor the peripheral serum cytokines (IL-6, IL-10, TNF*α*, sCD25, and IFN-*γ*), and chemiluminescence (ECL) was used to monitor ferritin. The laboratory monitoring was done on d0, d3, d7, d14, d21, and d28 and then monthly until 6 months after transfusion of CART. Thereafter, the monitoring was further continued every 3 months until 24 months after the transfusion. The response was assessed by computed tomography (CT) and contrast-enhanced magnetic resonance once per month within 6 months after CART, and positron emission tomography/computed tomography (PET/CT), enhanced magnetic resonance imaging (MRI), or positron emission tomography/magnetic resonance imaging (PET/MRI) every 3 months until 24 months after CART transfusion while CSF assessments are monthly for three months and then quarterly for up to 24 months. The efficacy was assessed by two lymphoma specialists independently according to Lugano criteria (2014) [40]. Progression-free survival (PFS) is defined as the time from enrollment to the date of disease progression or last follow-up or death from any cause. Overall survival (OS) is defined as the time from enrollment to the date of last follow-up or death from any cause.

In terms of treatment-related adverse reactions, cytokine release syndrome (CRS) and immune effector cell-associated neurotoxicity syndrome (ICANS) were graded according to the America Society of Transplantation and Cellular Therapy consensus criteria [41] and were treated according to Lee et al. [41]. In addition, anti-epilepsy drugs were also administered for seizure prophylaxis. Based on the National Cancer Institute CTCAE (Version 5.0), toxicities on organs were assessed. The assessment of engraftment of ASCT was as follows: a neutrophil count ≥0.5 × 10^9^/L for three continuous days was considered granulocyte engraftment, and a platelet count >20 × 10^9^/L for seven continuous days when no platelet infusion was performed was considered platelet engraftment.

Fluorescence in situ hybridization (FISH) was used to detect the amplification and ectopic rearrangements of *BCL2/BCL6/MYC* in tumor tissues. Next generation sequencing (NGS) was used to detect hotspot mutations in 225 lymphoma-related genes, where the sequencing depth was >1500x.

### 2.3. Statistical Analysis

SPSS 26.0 software and GraphPad Prism 9.0 software were used for statistical analysis. The chi-square (*χ*^2^) or Fisher test was used for the analysis of categorical data and the evaluation of associations between variables and efficacy. The Kaplan-Meier method was used for univariate analysis of progression-free survival (PFS) and overall survival (OS). The rank-sum test was used for the analysis of CART cell expansion. *P* < 0.05 was considered statistically significant.

## 3. Results

### 3.1. Clinical Characteristics

Baseline patient characteristics listed in Table 1 indicate that 17 CNS involvement patients, with a median age of 42 years (range of 19 to 66), comprised 9 (53%) males and 8 (47%) females. 15 (88%) patients had secondary CNS B-cell lymphoma (mantle cell lymphoma, *n* = 1; Burkitt lymphoma, *n* = 1; diffuse large B-cell lymphoma non-GCB, *n* = 9; and diffuse large B-cell lymphoma GCB, *n* = 4), and 2 (12%) had primary central nervous system B-cell lymphoma. All the patients were diagnosed with Ann Arbor stage IV. For 14 patients aged <60 years, the age-adjusted international prognostic index (aaIPI) ≥3 was 8, and for 3 patients aged ≥60 years, the international prognostic index (IPI) was 5, 4, and 4, respectively. Clinical symptoms and signs at the time of enrollment included headache 65% (11/17); blurred vision and diplopia 12% (2/17); nausea and vomiting 18% (3/17); convulsion 6% (1/17); waist pain, lower limb numbness, and reduced muscle strength 24% (4/17); hearing loss 12% (2/17); and distortion of the commissure 12% (2/17). The Eastern Cooperative Oncology Group performance status score ranged from 2 to 4 points. FISH assays for *MYC/BCL2/BCL6* in tumor tissues were performed in 11 (65%) patients. Two of them also received P53 measurements (Table 1). The abnormal factors involved *MYC/BCL2/BCL6* rearrangement and/or amplification and P53 deletion. Among them, 3 (patient No.1, patient No.7, and patient No.17) were diagnosed double hit lymphoma and one (patient No.3) had P53 deletion. Next-generation sequencing for gene mutation in tumor tissues was performed in 11 patients, and 9 patients had gene mutations positive, including TP53 (5/11), KMT2D (4/11), CD79b (3/11), CCND3 (3/11), CREBBP (2/11), TET2 (2/11), and MYD88 (1/11), as shown in Table 1. A total of 17 patients received ≥2 lines of antineoplastic therapies, and the median number of prior therapies was 11 (range of 5 to 18), as shown in Table 2. In 17 patients, 7 (7/17) were insensitive to chemotherapy and refractory, while the remaining 10 (10/17) patients had relapsed after first-line/second-line therapy, especially in combination with targeted drug therapy (BTKi, *n* = 9; BCL2 inhibitor, *n* = 8; and programmed death-1 inhibitor, *n* = 1). 3 (3/17) patients had progressed after ASCT, 5 (5/17) relapsed after CART therapy, and 6 (6/17) patients had a history of partial radiotherapy. At the time of enrollment, 5 (5/17) patients had isolated central nervous system involvement, and 12 (12/17) had systemic disease progression in addition to central nervous system involvement. Before the initiation of therapy, the disease status was progressive disease (PD) in 15 (15/17) patients and stable disease (SD) in 2 (2/17).

### 3.2. CART Transfusion and Dynamics

Among 17 patients, depending on the antigen expression of tumor tissue, 12 underwent CD19 CART cells (including 9 with murine-CD19 and 3 with humanized-CD19), with the median number of CART cells infusion of 1.44×10^6^ cells/kg (rang of 0.22×10^6^ cells/kg to 3.8×10^6^ cells/kg); 4 underwent hCD20 CART cells, with a median number of CART cells infusion of 1.29×10^6^ cells/kg (range of 0.94 × 10^6^ to 2.06 × 10^6^); and 1 underwent hCD22 CART cells, with infusion of 5.9×10^6^ cells/kg. The median peak number of CAR T-cell expansion was 163×10^6^ cells/L (range of 2.32 × 10^6^–920 × 10^6^) and achieved a peak with a median time of 9 days (range of 6 to 67) after CART transfusion, and the median lasting time of CART in peripheral blood was 31 days (range of 11 to 105). Three patients were infused with CART cells with a dose <0.5×10^6^ cells/kg because they had substantial disease burden. Patient No. 13 with Burkitt lymphoma treated by mCD19CART had abdominal bulky mass (13.3 cm × 9.1 cm × 13 cm) and brain parenchyma involvement, with infusion dose of 0.29×10^6^ cells/kg and peak number of 501 × 10^6^/L on +11 days and lasting for 33 days. Patient No. 14 treated by hCD19CART had abdominal bulky mass (8.7 cm × 7 cm) and brain parenchyma involvement, with infusion dose of 0.22×10^6^ cells/kg and peak number of 2.32 × 10^6^/L on +60 days and lasting for 105 days; and Patient No. 17 treated by mCD19CART had breast bulky mass (11 cm × 8.3 cm × 3.2 cm) and both brain parenchyma and CSF involvement, with infusion dose of 0.26×10^6^ cells/kg and peak number of 920 × 10^6^/L on +14 days and lasting for 53 days.

**Figure 3:**
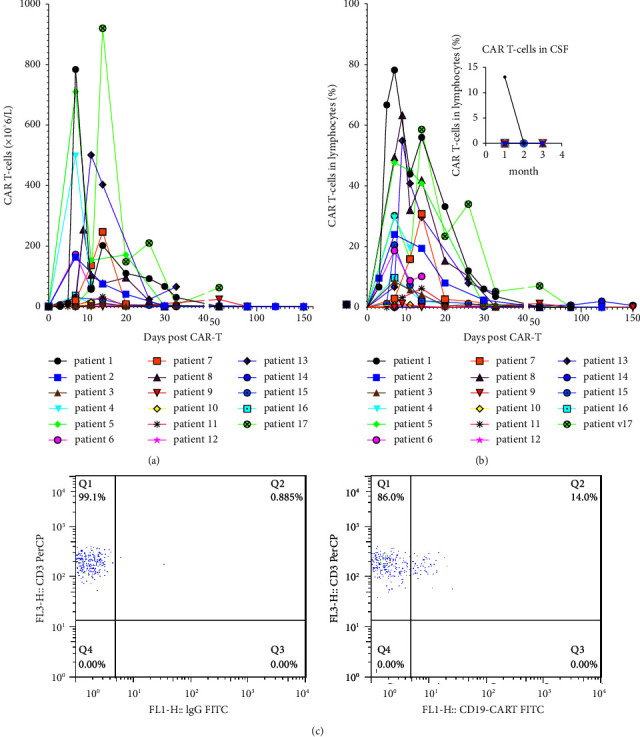
Dynamic changes of CART cells in peripheral blood and partial in cerebrospinal fluid after CART cell treatment. (a) Expansion and persistence of CART cells in peripheral blood were quantified by flow cytometry. (b) Percentage of CAR T-cells in lymphocytes(%) in peripheral blood and cerebrospinal fluid after CART cell therapy. (c) Detection of CART cells in the cerebrospinal fluid of patient No. 1 on day +30 by flow cytometry after infusion.

Out of 17 patients, lumbar puncture and CART cells in the CSF detection were performed in eight patients at the first month (Figure 3). CART cell trafficking into the CSF was noted in patient 1 when the number of CART cells in the PB was 92.6 × 10^6^ cells/L. While cells were not detected in the remaining 7 patients, the number of CART cells in PB dropped below the limit of detecting at that time.

The median number of CART infusion was 3.48×10^6^ cells/kg (range of 1.25 × 10^6^ to 5.9 × 10^6^) vs. 0.94 × 10^6^ cells/kg (range of 0.22 × 10^6^ to 1.9 × 10^6^) (*p* = 0.02), the median peak number of CART was 250.2 × 10^6^/L (range of 12.3 × 10^6^ to 784 × 10^6^) vs. 29.4 × 10^6^/L (range of 2.32 × 10^6^ to 960 × 10^6^) (*p* = 0.054), the median time to peak expansion was 7 days (range of 7 to 15) vs. 12 days (range of 6 to 68) (*p* = 0.16), and median lasting time of CART was 47.5 days (range of 11 to 112) and 33 days (range of 12 to 105) (*p* = 0.88) in the ASCT group and non-ASCT group, respectively.

### 3.3. Efficacy Assessment and Survival Analysis

Sixteen patients received bridging chemotherapy with the R-MA (4/16) and TEDDI (12/16) regimens to reduce the tumor burden prior to CART cell transfusion. At the time of infusion, all patients with CSF involvement had negative CSF by FCM, the symptoms and signs were managed, and the disease status was PD (*n* = 8), PR (*n* = 7), and CR (*n* = 2). 8 (8/17) patients underwent ASCT plus CART, and 9 (9/17) patients received CAR T-cell alone therapy, including 4 patients with single CART administration and 5 patients with short-interval sequential CD19/CD20/CD22CART treatment (within 3 months). The conditioning regimen before ASCT plus CART included the TEAM (75%) and non-TEAM (25%) regimens, and the median dose of CD34 cell transfusion was 2.35 × 10^6^/kg (range of 2 × 10^6^ to 5.2 × 10^6^). It was bendamustine (3/17) or fludarabine (5/17) monotherapy or in combination with cyclophosphamide (5/17) that was performed for lymphodepletion. Still, 4 patients (Patient No. 3, Patient No. 9, Patient No. 13, and Patient No. 16) did not undergo lymphocyte clearing because the absolute lymphocyte count was <0.2 × 10^9^/L. Taking the date of CART transfusion as day 0, ASCT was performed on day -1 in 5 patients, day -30 in 1 patient, and day -60 in 2 patients.

According to the three-month assessment after CART cell infusion, responses were observed in 12(12/17) patients and consisted of 11 CRs and 1 partial remission. One (1/17) patient with Burkitt lymphoma had a progressive disease with systemic and CSN involvement. Four (4/17) patients had progressive diseases with only systemic relapse, two of whom had a p53 gene mutation positive. Further analysis, the CRR was significantly higher in the ASCT group than in the non-ASCT group (100% vs. 44%, *p* < 0.01).

**Figure 4:**
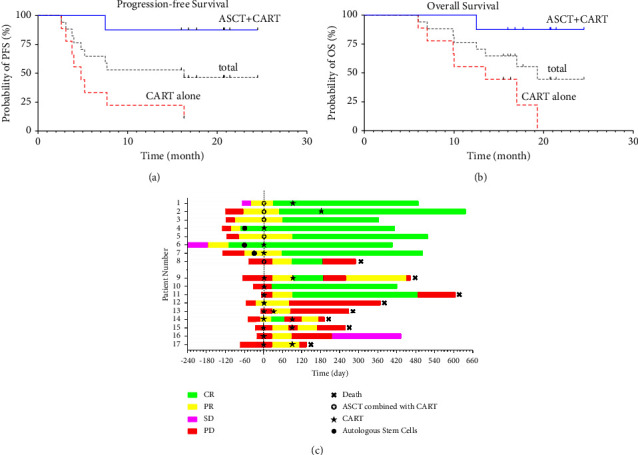
Therapeutic effect of CART treatment and duration of response, progression-free survival (PFS), and overall survival (OS) estimates. (a, b) Kaplan-Meier estimates of progression-free survival and overall survival. (c) Swimmer's plot of response for all patients on study (*n* = 17). Different colors represent the disease status. CR, complete remission; PR, partial remission; SD, stable disease; PD, progressive disease. Day 0 shows CAR T-cells infusion. ASCT, autologous stem cell transplantation; PFS, progression-free survival; OS, overall survival.

**Figure 5:**
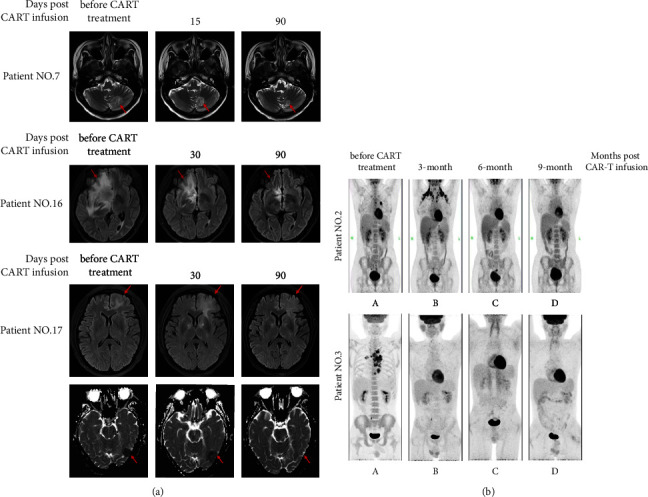
Pretreatment and post-treatment imaging. Representative MRI imaging before (left) and after (right) therapy (a). The main central invasion sites of patient No.7 are cerebellum vermis and hemispheres. Invasion sites of patient No.16 are corpus callosum and right frontal lobe. The central invasion sites of patient 17 are left occipital lobe and bilateral frontal cortex. The images of patient No. 2 and patient No 3 by PET/CT (b). MRI, magnetic resonance imaging; PET/CT, positron emission computed tomography.

By September 30, 2021, with a median follow-up of 20.7 months (range of 6 to 24.5), 8 (8/17) patients had achieved sustained remission. The median progression-free survival (PFS) of these challenging patients was 16.3 months (range of 2.6 to24.5 months). The eight patients with durable remission included seven patients treated by ASCT plus CART cells and one patient by CART cells alone (Figures 4 and 5). Disease progression occurred in the remaining 9 patients (1 in the ASCT group and 8 in the non-ASCT group), and the median time of progression of the 9 patients was 4.8 months (range of 2.6 to 16.3). For the 9 PD patients, 8 patients (1 in the ASCT group and 7 in the non-ASCT group) died, including 7 who died of disease progression and one (patient No. 9) who received allogeneic hematopoietic stem cell transplantation in the following treatment died of infection by CMV pneumonia. The median overall survival (OS) was 19.3 months (range of 6 to 24.5). Kaplan-Meier survival analysis showed that patients who underwent ASCT plus CART cells had longer PFS (*P* < 0.01) and OS (*P* < 0.01) (Figure 4). The median PFS and median OS in the ASCT group were not reached, while in the non-ASCT is 4.8 months (range of 2.6 to 16.3) and 13.5 months (range of 6 to 19.3).

Especially, further analysis of 9 patients who only received CART therapy showed that the median PFS and median OS of 5 patients with sequential different targeted CAR T-cell therapy were 4.8 months (range of 2.6 to 7.7) and 9.9 months (range of 6 to 17), and that of 4 patients who did undergo single targeted CAR T-cell infusion were 10.15 months (range of 3.1 to 16.3) and 15.9 months (range of 9.9 to19.3). For the three patients with double-hit lymphoma, two received ASCT plus CART treatment are in ongoing complete remission, while one with short-interval (within 3 months) sequential infusion of anti-CD19 and anti-CD20CART-cell died in 6 months after enrollment. For these 5 (5/11) patients with P53 gene mutation positive, the prognosis was worse (3 PDs, 1 PR, and 1CR) in three-month assessment after CART infusion, and by September 30, 2021, 3 died of progression diseases and the median OS is 10 months (range of 6 to 16). However, the one treated by ASCT plus CART was in durable remission. We did not find that the other gene mutations such as CD79b\KMT2D have a relationship with the prognosis due to the fewer number of cases. 

### 3.4. Toxic Effects

ICANS is the most concerning toxic effect of immunotherapy in r/r CNS lymphoma. In the 17 patients, 6 (35%) patients experienced ICANS, including grade 2 (*n* = 1), grade 3 (*n* = 2), and grade 4 (*n* = 3), and the median time of ICANS occurrence was 6 days (range of 1 to 8) after CART transfusion. The manifestations observed in patients were the following: headache, nausea, and vomiting in 5 patients (5/17), with a median onset of 7 days (range of 2 to 8) after CART; ataxia in 1 patient (1/17), where onset time was 3 days after CART; convulsion in 4 patients (4/17), where the median time of occurrence was 7.5 days (range of 5 to 23) after CART; coma in 3 patients (3/17), where the median time of occurrence was 8 days (range of 7 to 8) after CART; somnolence in 5 patients (5/17), where the median time of occurrence was 8 days (range of 3 to 8) after CART; and visual abnormalities in 2 patients (2/17), where the time of occurrence was 3 and 5 days after CART, respectively. After the intervention, the median duration of ICANS was 4.5 days (range of 3 to 23). The rate of ≥grade 3 ICANS was 29% (5/17). 3 patients developed grade 4 ICANS. Patient No. 8, who had previously underwent whole brain radiotherapy, had fever on d0 after CART cell transfusion. On d5, he suffered from neurological toxicity, which is manifested as hallucination, visual abnormality, somnolence, disorientation, and anomia; and on d24 after a CART transfusion, this patient was in a coma. DEX, mannitol, diazepam, and phenobarbital, which were initiated on d8, were administered for treatment, and the patient completely recovered on d40. Patient No. 17, who previously underwent radiotherapy for breast lymphoma, had a high fever that occurred on d2 after a CART cell transfusion and lasted for 5 days. The patient had neurological toxicity on day 7, and the manifestations included delirium and grand mal epilepsy. After treatment with mannitol, DEX, diazepam, and phenobarbital, the patient completely recovered on d28 after a CART cell transfusion. Patient No. 13, who had Burkitt lymphoma with bone marrow involvement, had a fever that occurred on d0 after CART transfusion and progressed to a high fever on d5, lasting for 4 days. Neurological toxicity occurred on d8, and the manifestations included convulsion of the limbs, urinary incontinence, and coma. After treatment with mannitol, DEX, sodium valproate, and diazepam, the patient completely recovered on day 12 after the CART cell transfusion. All severe ICANS in patients were alleviated, and neurotoxicity-related symptoms were reversible. No treatment-related deaths occurred in this study.

**Figure 6:**
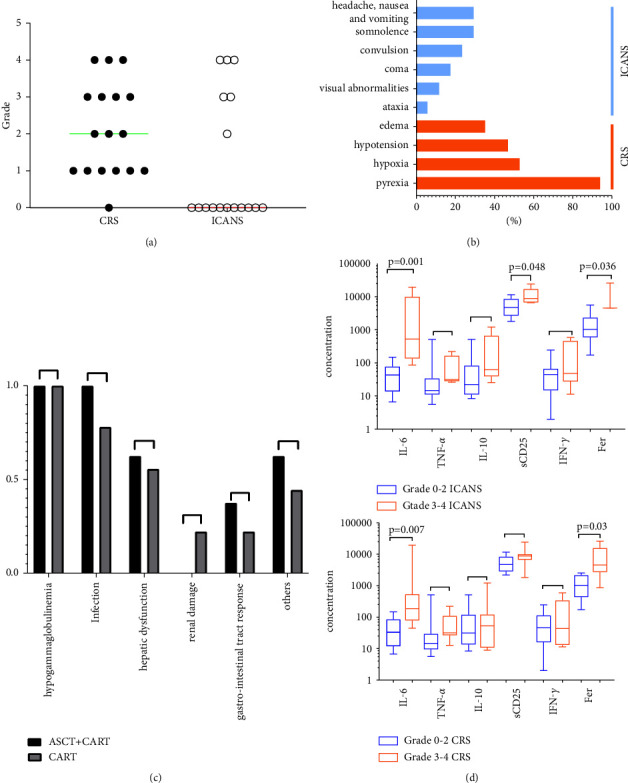
Adverse events associated with CART treatment. (a, b) Cytokine release syndrome (CRS) and immune effector cell-associated neurotoxicity syndrome (ICANS). (a) The grade of cytokine release syndrome- and immune effector cells-associated neurologic toxicity in all patients, and the horizontal line indicates the median. (b) The rate of each symptom of CRS and ICANS in all patients. (c) There are no differences in complications between the ASCT plus CART group and CART alone. (d) Severe ICANS (≥grade 3) has association with IL-6, IFN-*γ*, and ferritin. Severe CRS (≥grade 3) was related with IL-6 and ferritin.

CRS is another common adverse reaction to CART cell immunotherapy. It occurred in 16 patients (94%), and the median time of CRS occurrence was 1 day (range of 1 to 8) after CART transfusion. The major manifestations included the following: pyrexia in 16 patients (94%), with the median time of occurrence of 1 day (range of 1to8) after CART transfusion; hypotension in 8 patients (8/17), where the median time of occurrence was 3 days (range of 2 to 9) after CART transfusion; hypoxia in 9 patients (9/17), where the median time of occurrence was 5 days (range of 2 to 15) after CART transfusion; and generalized edema in 6 patients (6/17), where the median time of occurrence was 3 days (range of 2 to 8) after CART transfusion (Figures 6(b)). The median duration of CRS was 10 days (range of 4 to 29) after CART transfusion when corresponding interventions were performed. Grade 3 or higher CRS was observed in 7 (41%) patients, three of whom (patient No. 8, patient No. 16, and patient No. 17) received radiotherapy; three of whom (patient No. 1, patient No. 13, and patient No. 17) had bone marrow involvement; and four of whom (patient No. 1, patient No. 3, patient No. 5, and patient No. 8) had underwent ASCT + CART. Specifically, the incidence of ≥grade 3 CRS was 50% and 33% (*p* = 0.48) and of ≥grade 3 ICANS was 25% and 33% (*p* = 0.14) in the ASCT and non-ASCT groups, respectively.

The common adverse events in the treatment period included agranulocytosis (17/17), infection (15/17), hypogammaglobulinemia (17/17), hepatic dysfunction (12/17), abnormal renal function (2/17), and gastrointestinal hemorrhage (3/17) (Figure 6). None of the patients received supportive therapy with growth factors. High-intensity conditioning in the ASCT group did not significantly increase the duration of agranulocytosis (13.38 ± 5.85 days vs. 15.78 ± 6.63 days, *p* = 0.65). The time of neutrophil cell engraftment was 11 days (range of 10 to 30), and platelet engraftment was 12 days (range of 10 to 14) in patients who underwent ASCT plus CART cells, which was consistent with previous findings [42–44]. These results indicated that CART cells did not influence the engraftment of hematopoietic stem cells.

**Figure 7:**
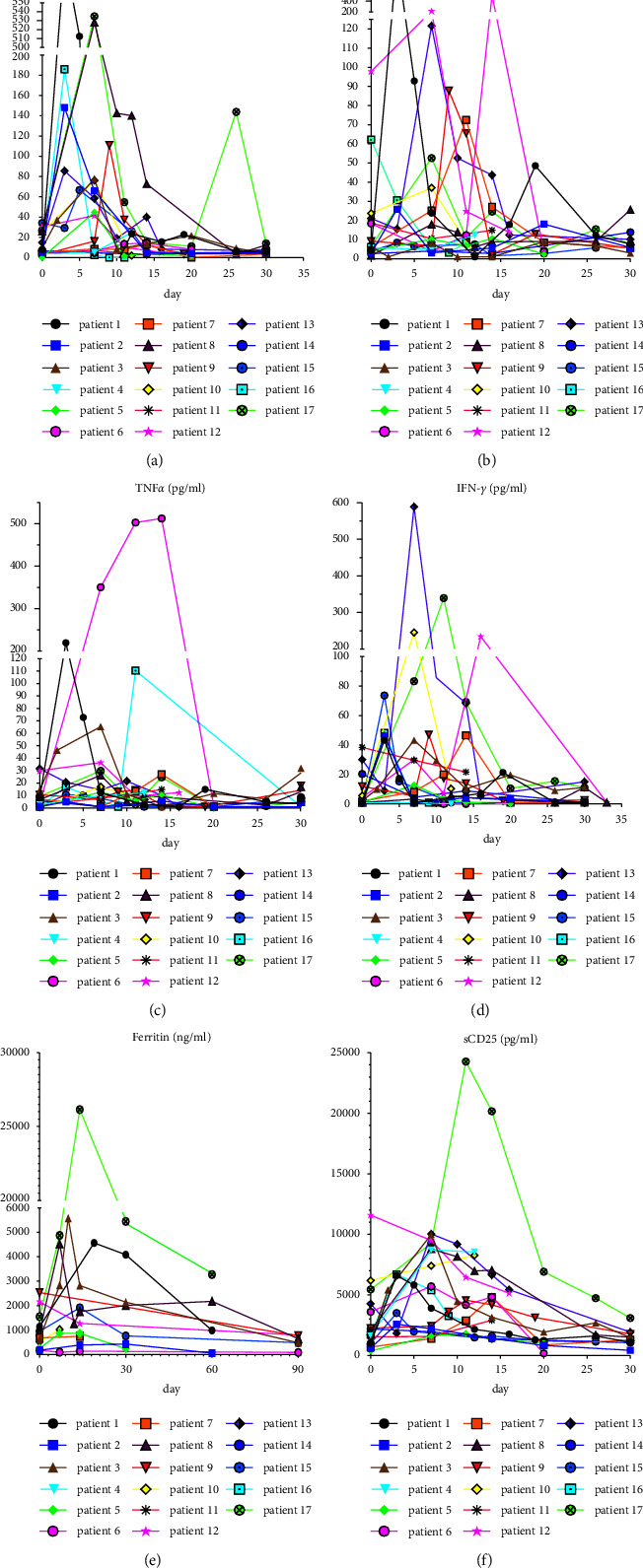
Changes of indicators during CART cell therapy. (a–f) IL-6, IL-10, TNF*α*, IFN-*γ*, ferritin, and sCD25 levels of the 17 patients during CART cell therapy. The day of first CAR T-cells infusion was day 0.

The changes in cytokines (IL6, TNF*α*, IL10, sCD25, and IFN-*γ*) and ferritin are shown in Figure 7. The median peak time was 7 days (range of 0 to 14), 7 days (range of 0–14), 7 days (range of 0–30), 7 days (range of 0–14), and 7 days (range of 0 to 30) after CART-cell transfusion, respectively. The median levels of IL-6 and ferritin were 76.15 ng/ml (range of 6.67 to 19540) and 2037.25 ng/ml (range of 172.8 to 26143.9), respectively. The severity of ICANS was positively correlated with IL-6 and ferritin levels (Figure 6).

## 4. Discussion

Considering that patients with r/r CNS lymphoma have a short survival time and a poor prognosis [10–13], no effective treatment is currently available for it. Over recent years, although CAR T-cell immunotherapy has been demonstrated effective and safe for r/r CNS B-cell lymphoma by several case reports, series, and studies [20–24, 26], disease progression can occur shortly after treatment [25, 26]. Therefore, attempts have been made to explore options for prolonging PFS: one study held that CAR T-cell therapy following ASCT had a long-term response with a median PFS of 14.03 months [33]. While one reported that patient with dual CD19/CD70 CART therapy attains remission lasting for 17 months [20]. Nonetheless, limited data compared the impact of ASCT plus CART versus sequential CD19/CD20/CD22 or targeting other tumor antigen CAR T-cell therapy on advanced r/r CNS lymphoma. In addition, most previous studies were in overall low sample size. This study is a larger sample size for the investigation of the safety and effectiveness of CART cells in the treatment of advanced r/r CNS lymphoma and firstly compared the impacts of ASCT plus CART cells versus short-interval sequential CAR T-cell therapy on sustained remission.

The overall response rate (ORR) was 71% (12/17), and the complete remission rate (CRR) was 65% (11/17) at 3 months after CART cell transfusion in our study, which was similar to the CRR in relapsed/refractory B-cell lymphoma patients without CNS involvement who underwent CART cell therapy (58%) [14, 16, 26, 45]. The median PFS of the 17 patients was 16.3 months, and 9 patients (including 7 in the ASCT plus CART group and 2 in the CART group) had a PFS >1 year. 29% (5/17) of patients experienced disease progression, with the median time of PD was 3.8 months (range of 2.6 to 5.2 months). Three of these five patients with PD had a p53 gene mutation-positive, as previous findings report that these patients belong to a population with a poor prognosis and resulted in a nonresponsive outcome [9, 46]. However, in our study, other gene mutations had not been found in correlation with prognosis due to a smaller sample size.

In addition, further analysis showed that the remission rate was significantly higher in the ASCT group than in the non-ASCT group, and that the duration of PFS was longer. We speculated that the observed differences could be due to the following: (1) high-dose chemotherapy prior to transplantation could reduce tumor volume and induce remission in patients, while lymphocyte clearing was more complete, which could favor the implantation of adaptive immune cells, enhance the expansion of adoptive T cells, and improve antitumor effects, namely, hematopoietic stem cell-driven lymphocyte proliferation [47–49] and especially the proliferation of CD8^+^ T cells [49–52]; (2) high-dose conditioning chemotherapy could clear implantation-inhibitory substances in the lymphoma microenvironment, improve the tumor immunosuppressive microenvironment (TME) [53–56], and favor CART cells to kill tumor cells and promote the infiltration of CART cells in tumor tissues. In addition, the treatment regimen ASCT plus CART, i.e., HSCT followed by CART transfusion, could maintain a relatively long duration of sustained remission, which could be associated with the fact that CART cells could purify possibly contaminated autologous hematopoietic stem cells for transplantation, thus effectively reducing the risk of relapse (Figure 5).

Interestingly, the prognosis of the three double-hit lymphoma seemed not very bad in our study. 2 (2/3) patients with double-hit lymphoma who received ASCT + CART therapy are in ongoing remission until the cutoff date. Because of the small number of cases, we did not yet conclude that combination therapy is expected to improve the poor outcome of the double strike. However, this is promising. Another attractive phenomenon is that contrary to a previous study (see [20, 45, 57]), for these 9 patients with CART cell therapy alone, we found that the median PFS in 5 patients who underwent sequential CAR T-cell infusion was not better than that in 4 patients who received a single CD19/20/22CART administration, and neither was the OS. These findings demonstrated that sequential CART cells did not benefit patients with early relapse after CART cells. It appears that sequential infusion of CART-cells is not superior to single CAR T-cell treatment for some patients, and it is essential for screening of these patients. Whether it is necessary to sequentially administrate the second or the third different CART cells for a longer durable response, a prospective study with a larger sample size is needed to design, and the further relationship needs more investigation.

Flow cytometry was used to monitor CART in this study. Like previous findings [14, 16, 45], the median peak time of CART cell expansion was within 2 weeks in the 17 patients, and the median duration of CART cells in peripheral blood was 31 days. Even in patients with sustained remission, CART cells were not detected, indicating that long-term efficacy may not require the persistent expansion or presence of CART cells, which needs to be further investigated in future studies. In addition, this study also showed that CART cell expansion peaked on day 67 after transfusion in Patient No. 14, who was treated with hCD19CART, lasting for 105 days, but this patient also had short-term disease progression, which indicated that human derived CART cells had longer persistence in vivo.

After CART cells infusion, CSF was examined in 8 patients. CART cells in CSF were detected by FCM in patient No. 1, indicating that CART cells could pass the blood-brain barrier (BBB). However, CART cells in CSF were not detected in the remaining 7 patients, which may be associated with lumber puncture, and CSF assessments were not done at earlier days of the CART treatment due to concerns for hypersive intracranial pressure resulted by ICANS. At one month or later after the infusion when patients have passed the crisis, CSF assessment was performed, and meanwhile, the expansion peak of CART cells was dropped. Most of them (7/8) even lower the detectable threshold of quantification of technology in peripheral blood. Safety is an essential precondition for CSF detection. Moreover, patients without CSF-CART detection had good outcomes . The detection of CART in CSF has not been suggested as a clinical routine test (Figure 3).

Repuncture was performed for relapsed patients (Patient No. 9 and 15, both of whom underwent simple CART therapy) to acquire CSF or tumor tissues for FCM, which showed that the target antigen was still expressed. Contrary to previous studies [26], no CART cells were found in the CSF of the patients, and CART did not appear with the target antigen positive tumor cells. In addition, the CART counts were lower, and the sustained time was shorter in CSF than in peripheral blood, which could be associated with the intracranial immunosuppressant environment.

CRS and ICANS are common toxic effects of CART therapy. For patients with r/r CNS lymphoma, the incidence and severity of ICANS are of greater concern. In this study, the incidence of ≥grade 3 ICANS was 29%, which was higher than that of other studies in the noncentral nervous system lymphoma (10%, 12%) [47, 48] but was comparable to the incidence of neurotoxic effects reported in previous studies on CART therapy for CNS lymphoma (ranging from 32% to 40%) [25, 26, 33]. No elevated ICANS incidence or lethal neurotoxicity occurred, all the ICANS symptoms were reversible, and no treatment-related deaths occurred in this study.

The dose range of CAR T-cell infusion was wide (from 0.22×10^6^ cells/kg to 5.9×10^6^ cells/kg). Based on concurrent systemic lymphoma, most patients received conventional dose of CART cell infusion, except forthree patients. According to previous studies, patients with a substantial disease burden, in particular those with rapidly progressive disease and/or bulky extramedullary disease, are at risk of severe ICANS. Apart from that, the severe ICANS is associated with CART cell peak expansion and dose of infusion [58–60]. To reduce the incidence of severe neurotoxicity, three patients (Patient No.13, Patient No.14, and Patient No.17) with high disease burden in our study received fewer infusion dose (<0.5×10^6^ cells/kg). However, the expansion peak and persistence of CART cells in these three patients were not affected, and two of them suffered from grade 4 ICANS (one without ICANS may be associated with humanized CAR T-cell therapy). Further analysis demonstrates that infusion dose has no relevant to the occurrence and severity of neurotoxicity but to the efficacy of the treatment. Due to the small sample size, further research is needed.

In the present study, the incidence of ≥grade 3 CRS was 41.17%, which is higher than the results reported in other studies (22%) [15]. It may be associated with conditioning chemotherapy deeply lymphodepleting and enhance to the expansion of CART cells. Three(Patients No. 8, Patients No.13, and Patients No.17) had grade 4 ICANS and CRS, where Patient No. 8 had previously undergone whole-brain radiotherapy, and patient 17 had undergone radiotherapy for the primary tumor (breast involvement). Consequently, these findings could be associated with the destruction of the tumor microenvironment by radiotherapy and the “abscopal effects” [61, 62]. Cytokines and ferritin were positively correlated with the severity of ICANS, which was in line with previous studies [16, 63, 64]. Dynamic monitoring of the cytokine spectrum (IL6, TNF*α*, IL10, sCD25, and IFN-*γ*) and ferritin showed that cytokine levels increased with the expansion of CART cells. Our results also showed that the incidence of ≥grade 3 ICANS and CRS was not significantly different between the ASCT plus CART versus CART alone group, indicating that ASCT plus CART combination therapy does not increase the inflammatory toxicity and neurotoxicity of CART.

The 17 patients all had different degrees of hypogammaglobulinemia, which could be associated with poor B-cell hyperplasia. Comparing the ASCT plus CART group versus the non-ASCT group showed that high-intensity chemotherapy did not increase in infection or prolong the duration of agranulocytosis in patients. No growth factor was used for supportive therapy in treatment, and the adverse events did not significantly differ between the ASCT group and the non-ASCT group.

The comparison between the ASCT and non-ASCT groups showed that the remission rate was higher and PFS/OS was longer in the ASCT group, while the incidence of severe ICANS and CRS was comparable between the two groups. In addition, CART cells in the ASCT group did not influence transplantation, and high-intensity conditioning for transplantation did not prolong the duration of agranulocytosis or increase the incidence of infection. These findings have an important referencing significance for designing treatment strategies for r/r CNS lymphoma as they could provide a new treatment regimen for r/r CNS lymphoma. However, the sample size of this study was relatively small, the follow-up time was relatively short, and the grouping was not randomized. As many clinical factors were involved in the grouping, there could be a bias at baseline. Therefore, more multiple-center studies with longer follow-up times are needed for further investigation. Finally, our findings show that ASCT plus CAR T-cell therapy could be the most effective treatment for r/r CNS B-cell lymphoma but still have higher severe ICANS in CNS lymphoma patients than in non-CNS lymphoma patients. Therefore, CART cells should be applied with caution in the treatment of r/r CNS lymphoma.

## Figures and Tables

**Figure 8:**
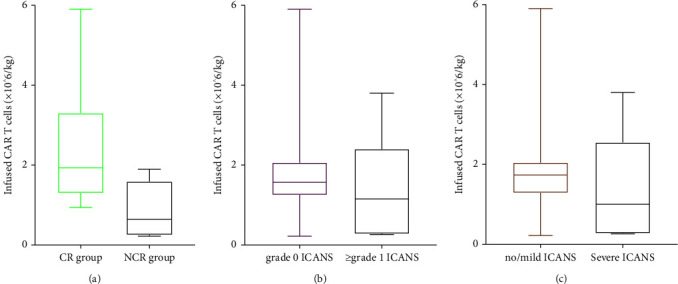
(a) The correlation of dosages of CAR T-cell infusion with the occurrence of complete response (*p*=0.02). (b, c) The correlation of dosages of CAR T-cell infusion with the occurrence/severity of ICANS (*p*=0.36 and *p*=0.20, respectively). Horizontal lines indicate medians.

**Table 1:** Clinical characteristics of patients.

ID	Sex	Age	Disease pathology	Stage	aaIPI/IPI	PCNSL	Site of CNS disease maximal dimension (mm)	Tumor in CSF (%)	Tumor in BM (%)	NGS	FISH	Previous CART therapy	Previous ASCT therapy	Disease status
1	F	39	DLBCL GCB	IVB	2	N	CSF	28%	60%	Negative	MYC/BCL2 rearrangement	N	N	SD
2	F	32	DLBCL non-GCB	IVB	2	N	Cerebellum, left frontal lobe (4)	N	N	^*∗*^NA	^*∗*^NA	N	N	PD
3	M	34	DLBCL non-GCB	IVB	3	N	T3-5 thoracic cord (17)	N	N	TP53 p.W146X; STAT3 p.E616del; TET2 p.Q916X p.R1452X; CD79B p.E185X; CHD8 p.R986X; NFKBIE p.Y254Sfs^*∗*^13; BCL10 p.I46Yfs^*∗*^24,	TP53 deletion. BCL6 rearrangement	mCD19CART(PR) hCD22CART(PD)	N	PD
4	M	48	DLBCL non-GCB	IVA	2	N	Pons, right thalamus	N	N	^*∗*^NA	^*∗*^NA	N	N	PD
5	M	66	DLBCL non-GCB	IVB	5	N	Bilateral paraventricular, basal ganglia (4)	N	N	^*∗*^NA	MYC/BCL6/BCL2 amplification	N	N	PD
6	M	43	DLBCL non-GCB	IVA	2	Y	Left frontal lobe, basal ganglia	N	N	^*∗*^NA	^*∗*^NA	N	N	SD
7	F	42	DLBCL GCB	IVA	3	N	Cerebellum vermis and hemispheres (41.4)	N	N	^*∗*^NA	MYC/BCL2 rearrangement. BCL6 amplification	N	YES	PD
8	F	47	DLBCL Non-GCB	IVB	3	Y	Right frontal lobe	N	N	MYD88 p.L265P; CD79B p.Y196C; KMT2D p.R1702X; ETV6 p.Q7Afs^*∗*^54; CCND3 p.Q280X; PIM1 p.S189Vfs^*∗*^20; CDKN2A p.A13Lfs^*∗*^13;	BCL6 rearrangement. MYC/BCL2 amplification	N	N	PD
9	M	40	DLBCL non-GCB	IVA	2	N	CSF	7.47%	N	TNFAIP3 p.Q74X; PRDM1 p.L48Vfs^*∗*^5; CDKN1B p.L144X; CCND3 p.D286Lfs^*∗*^72; CARD11 p.R337Q; PCLO p.Q3300X; NUDT15 p.R139C	BCL6 rearrangement	mCD19CART(PD) hCD22&CD19CART(PR)	N	PD
10	M	41	DLBCL non-GCB	IVA	2	N	CSF	21.11%	^*∗*^NA	KMT2D p.Q3915X; CD70 p.Q47X	^*∗*^NA	mCD19-CART(CR)	N	PD
11	M	58	DLBCL non-GCB	IVA	3	N	Lumbar cord (90 mm)	N	2.77%	IRF4 p.K123R	Negative	N	YES	PD
12	M	64	MCL	IVB	4	N	CSF	16.13%	20.50%	Negative	^*∗*^NA	N	N	PD
13	F	19	BL	IVA	3	N	Bilateral occipital lobe, left frontal lobe	N	93.5%	TP53 p.R213X, FOXO1 p.S203R, ID3 p.L40Efs^*∗*^21, TET2 p.E1151X; MYC p.A59T, CCND3 p.T283A	^*∗*^NA	N	N	PD
14	F	64	DLBCL non-GCB	IVB	4	N	Cerebrum	N	N	^*∗*^NA	Negative	mCD19CART(CR)	N	PD
15	M	33	DLBCL GCB	IVB	3	N	T4-6 thoracal cord	63.32%	N	TP53 p.N131Y; TNFRSF14 p.M1V; KMT2D p.W315X; CREBBP p.Q2118Sfs^*∗*^25; DDX3X p.K13OIfs^*∗*^3; RB1 p.Y790X; PTEN p.V191Sfs^*∗*^11	BCL2/BCL6 rearrangement. MYC amplification;	N	N	PD
16	F	35	DLBCL non-GCB	IVA	3	N	Corpus callosum, right frontal lobe (20 mm)	6.1%	10%	TP53 c.743G > A, BIRC3 p.R411K, DNMT3A p.R882C, KMT2C p.T2941	Negative	hCD22CART mCD19CART	YSE	PD
17	F	50	DLBCL GCB	IVA	3	N	Left occipital lobe, bilateral frontal cortex, (22 mm)	18.97%	NA	TP53 p.G245S; CD79B p.Y196S; CREBBP c.3837-2A > G; KMT2D p.A2119Lfs^*∗*^25; KMT2C p.C359Vfs^*∗*^15; ZMYM3 p.Q175Rfs^*∗*^52	MYC/BCL2 rearrangement BCL6;	N	N	PD

M, male; F, female; aaIPI, age-adjusted International Prognostic Index; IPI, International Prognostic Index; PCNSL, primary central nervous system lymphoma; CNS, central nervous system; DLBCL, diffuse large B-cell lymphoma; GCB, germinal center (GC)-like B-cell type; MCL, Mantle cell lymphoma; BL, Burkitt lymphoma; CSF, cerebrospinal fluid; ^*∗*^NA, not available; NGS, next generation sequencing; FISH, fluorescence in situ hybridization; tumor in CSF (%), percentage of B lymphoma cells in nuclear cells of cerebrospinal fluid; tumor in BM (%), percentage of B lymphoma cells in nuclear cells of bone marrow; CART, chimeric antigen receptor T cell; ASCT, allogeneic stem cell transplantation; PD, progressive disease; SD, stable disease.

**Table 2:** Treatment and effect of CART cell therapy.

ID	Primary treatment	Conditioning regimen	Lymphodepletion	Infused cells (10^^6^/kg)		CRS grade	ICANS grade	Neurologic toxicity	ICANS treatment
				CD34^+^	CART				
1	RCHOP × 3 (PD); isolated CNS relapse; *R* + HD-MTX + temozolomide + BCL2-inhibitor × 2 (SD)	TEAM	Bendamustine	5.2	mCD19 (3.8)	3	3	Angulus oris convulsion	Mannitol, glucocorticoid, sodium valproate
2	R-DA-EPOCH × 4 (PR); GVD + PD-1 inhibitor × 2 (PD); isolated CNS relapse; BTKi + HD-MTX + GVD + PD-1 inhibitor × 2 (PD) systemic disease progression and CNS involvement	BEAM	F	2.23	mCD19 (1.25)	1	0	None	Mannitol
3	R2-CHOPE (PD); spinal cord involvement; R-MT(SD); R-CHOPE + BCL2-inhibitor (PR); HD-MTX + R-CHOPE × 4 (PD); mCD19CART (PR); hCD22CART (PD); systemic disease progression and CNS involvement	BuCy	——	2.34	hCD20 (2.06)	3	0	None	None
4	EPOCH × 6 (CR); isolated CNS relapse; HD-MTX + DEX × 2 (PR); isolated CNS relapse. HD-MTX + Idarubicin + DEX (PD); DHAP × 2 (PD); MIDD + BTKi × 6 (PD)	TEAM	Bendamustine	3.22	hCD22 (5.9)	2	0	None	None
5	RCHOP × 3 (PR); RCHOP × 2 (PD); Isolated CNS relapse; MTX + BTKi + Temozolomide × 4 (CR); isolated CNS relapse (PD)	TEAM	F	2.37	hCD19 (3.3)	3	0	None	Mannitol
6	*R* + HD-MTX × 4 (PR); isolated CNS relapse; radiotherapy (PR); systemic disease progression and CNS involvement; ifosfamide + Ara-C × 1 (PD); HD-MTX × 2 (SD); TEDDi-R × 4 (PD); BTKi + BCL2-inhibitor × 4 (SD)	TEAM	FC	2	mCD19 (1.93)	1	2	ICE score 4; awakens to voice	Sodium valproate
7	RCHOP × 3 (PR); RCHOPE × 4 (CR); ASCT (CR) *R* × 4 (CR); isolated CNS involvement; *R* + MTX + temozolomide + BTKi (PD)	TEAM	FC	2	mCD19 (2)	2	0	None	Mannitol
8	WBRT (CR); systemic disease progression and CNS involvement; HD-MTX + RCHOP × 3 (CR); systemic disease progression and CNS relapse; HD-MTX + RCHOP × 2 (PD)	TEAM	Bendamustine	3.23	mCD19 (1.3)	4	4	Coma, seizures, hallucinations	Mannitol, glucocorticoid, levetiracetam, diazepam
9	R-CHOPE × 6 (CR); radiation therapy (CR); systemic disease progression. RICE(PD); R2GDP (PD); RDHAP (SD); r-DA-EPOCH(PR); CD19CART (PD); BTKi + radiation (SD); RICE (SD); CD22CART + CD19CART (PR); BCL2-inhibitor + Chidamide (PD) testicular relapse + CNS involvement	——	——	——	hCD20 (1.57)	1	0	None	None
10	RCHOP × 6 (CR); intraocular relapse (PD); RCHOP; radiation therapy (PD) RDHAP × 2 (PD); CD19-CART (CR) systemic disease progression and CSF involvement (PD)	——	F	——	hCD20 (0.94)	1	0	None	Mannitol, glucocorticoid
11	*R*2 + CHOP × 6 (CR); systemic disease progression; REPOCH × 6 (CR); BEAM + ASCT (CR) systemic disease progression and CNS involvement (PD); HD-MTX + DEX (PD); REPOCH (PD); RGDP (PD); MINE (PD)	——	FC	——	hCD19 (1.4)	1	0		Mannitol, glucocorticoid
12	RCHOP(PD); RDHAP (SD); BTKi + CHOP (SD); BTKi + DHAP (SD); BTKi + BCL2-inhibitor (PD) EPOCH (PD); GemOx × 2 (PD) systemic disease progression + CSF involvement	——	FC	——	mCD19 (1.485)	1	0	None	None
13	EPOCH × 2 (PD); decitabine + EPOCH × 2 (PD) COPADM (PD) systemic disease progression and CSF involvement	——	——	——	mCD19 (0.29)	3	4	Coma, seizures	Diazepam, mannitol, glucocorticoid, plasmapheresis
14	RCHOP × 6 (CR); systemic disease progression; R-DICE × 6 (CRu); systemic disease progression; mCD19CART (CR); systemic disease progression and CNS involvement (PD)	——	FC	——	hCD19 (0.22)	0	0	None	None
15	RB × 6 (CR); systemic disease progression; R-CHOP (PD); REDOCH × 4 (PD); RDHAP + BCL2 inhibitor (PD)CSF involvement + systemic disease progression.	——	F	——	mCD19 (1.9)	2	0	None	Mannitol, glucocorticoid
16	RCHOP × 4 (PR); RCHOP × 2 (CRu); isolated CNS involvement; RCODOX-M × 2; RCDOP × 4 (CR); BEAM + ASCT (CR); CSF + CNS involvement; radiation (PD); *R* + MTX(PD)*R* + MTX + BTKi (PD); MTX + temozolomide + VP-16;CD22CART + CD19CART + BCL2-inhibitor (PD)	——	——	——	hCD20 (1.0)	3	3	ICE score 2; Awakens only to tactile stimulus; dystaxia	Mannitol, glucocorticoid
17	RCHOP × 4 (PR); REPOCH (PD); RGDP(PD); RDICE × 2 (PR); RDICE × 2 + BCL2-inhibitor (PD) + BTKi + GemOx(PD); Radiation therapy + Chidamide + lenalidomide (PD); systemic disease progression and CNS involvement (PD)	——	F	——	mCD19 (0.26)	4	4	Coma; seizures	Mannitol, Glucocorticoid, Diazepam, Sodium valproate

CRS, cytokine-release syndrome; ICANS, immune effector cells associated neurologic toxicity syndrome; CHOP, cyclophosphamide, doxorubicin, vincristine, dexamethasone; HD-MTX, high-dose methotrexate; DA-EPOCH, dose adjusted etoposide, prednisone, vincristine, cyclophosphamide, doxorubicin; GVD, gemcitabine, vinorelbine, liposomal adriamycin; CHOPE, cyclophosphamide, doxorubicin, vincristine, dexamethasone, etoposide; DEX, dexamethasone; DHAP, dexamethasone, cytarabine, cisplatin; MIDD, methotrexate, ifosfamide, liposomal Adriamycin, dexamethasone; TEDDi, temozolomide, etoposide, liposomal adriamycin, dexamethasone, intrathecal injection(Ara-C); R2, rituximab, lenalidomide; ASCT, autologous stem cell transplant; WBRT, whole brain irradiation treatment; ICE, ifosfamide, carboplatin, etoposide; DICE, dexamethasone, ifosfamide, carboplatin, etoposide; MINE, mitoxantrone, ifosfamide, etoposide; GDP, gemcitabine, dexamethasone, cisplatin; COPADM, vincristine, high-dose methotrexate, doxorubicin, cyclophosphamide, prednisone; RB, rituximab, bendamustine; GemOx; gemcitabine, oxaliplatin; CODOX-M, cyclophosphamide, vincristine, doxorubicin, methotrexate; TEAM, thiotepa, etoposide, cytarabine, melphalan; BEAM, carmustine, etoposide, cytarabine, melphalan; BuCy, busulfan, cyclophosphamide; *F*, Fludarabine; FC, Fludarabine, cyclophosphamide; BTKi, Bruton's tyrosine kinase inhibitor; PD-1 inhibitor, programmed death-1 inhibitor; CNS, central nervous system.

## Data Availability

The datasets used and/or analyzed during the current study are available from the corresponding author on reasonable request.
